# Spatial–temporal evolution patterns of influenza incidence in plateau regions from 2009 to 2023

**DOI:** 10.3389/fpubh.2025.1553715

**Published:** 2025-04-02

**Authors:** Sheng-lin Qin, Hai-jun Bai, Ping Deng, Yi-wen Wang, Song-ming Ma, Yang Zhang, Yu-qi Jiang, Jiang Long, Jin-hua Zhao

**Affiliations:** ^1^Qinghai Provincial Center for Disease Control and Prevention, Xining, China; ^2^Department of Public Health, Medical College of Qinghai University, Xining, China; ^3^Disease Prevention and Control Institute, China Railway Qinghai-Tibet Group Co., Ltd., Xining, China; ^4^Chongqing Center for Disease Control and Prevention, Chongqing, China

**Keywords:** plateau region, influenza, incidence rate, spatiotemporal analysis, GIS

## Abstract

**Objectives:**

This study used (Geographic Information System) GIS technology to analyze the spatiotemporal distribution of influenza incidence in Qinghai from 2009 to 2023, based on influenza surveillance data.

**Methods:**

This study first accessed the influenza data sets of Qinghai Province from 2009 to 2023 through the Chinese Infectious Disease Surveillance System. Subsequently, trend charts of influenza incidence in each city and prefecture were employed to illustrate the trends of influenza incidence during the period from 2009 to 2023. To explore the risks of influenza incidence in different counties and districts, methods including spatial autocorrelation, cluster analysis, hotspot analysis, Gravity center shift model, and standard deviation ellipse were utilized.

**Results:**

The study showed that the incidence of influenza showed significant fluctuations, with marked spikes in 2019 and 2023. Spatial autocorrelation analysis revealed significant positive autocorrelation in 2015, 2017–2019, and 2022–2023 (Moran’s *I* > 0 and *p* < 0.05). Local spatial autocorrelation analysis identified clustering patterns in different regions, with high - high clustering in eastern Qinghai and low - low clustering in the west. Hot - spot analysis indicated that the counties with a high incidence of influenza in Qinghai were mainly located in the lower - altitude east. Standard deviation ellipse analysis showed that in 2021, the spread of influenza was the most extensive, almost covering eastern and parts of western Qinghai. From 2021 to 2022, the spread range shrank and expanded again in 2023. The gravity center of influenza moved southeastward year by year from Gangcha County in 2018 to Gonghe County in 2023. The spread of influenza was found to be expanding eastward, with the epidemic center shifted over time.

**Conclusion:**

The prominent spatiotemporal heterogeneity of influenza incidence in Qinghai Province indicates the need to develop differentiated and precise influenza prevention and control strategies in different regions to address the changing trends of influenza epidemics.

## Introduction

Influenza is a highly contagious viral infection that affects people of all ages. It is associated with high mortality rates during pandemics, epidemics, and sporadic outbreaks (1). The influenza epidemics’ rapid spread and extensive impact present substantial threats to public health and societal stability (2). Based on the data provided by the World Health Organization (WHO), globally, influenza affects approximately 10% of adults and 20% of children each year, leading to around 1 billion cases, 3–5 million hospitalizations, and up to 650,000 deaths annually (3, 4), thus bringing about a heavy disease burden and economic losses. Influenza is a global challenge, and future pandemics of influenza are inevitable.

Globally, influenza epidemics display a clear seasonal characteristic, where winter and spring typically constitute the peak periods of influenza occurrence (5). In China, due to the large population, vast territory, and diverse climates, influenza leads to an average of 88,000 additional respiratory deaths annually and the seasonality of influenza activity differs among various regions (6). In China, influenza activity varies regionally due to diverse climates and geographical factors. Qinghai Province, a typical plateau region, presents unique challenges due to its high-altitude climate and geographical isolation.

Geographic Information Systems (GIS) are powerful tools that facilitate the spatial analysis of health data (7). GIS presents benefits in spatiotemporal analysis, supplying novel perspectives and approaches for the surveillance and containment of influenza epidemics (8). Leveraging GIS technology enables the spatial visualization and quantitative analysis of influenza surveillance data, facilitating a more intuitive comprehension of the spatiotemporal distribution patterns of epidemics and uncovering disease transmission routes and influencing factors. In this study, GIS was used to assess the distribution and evolution of influenza incidence in Qinghai.

Although previous studies have analyzed influenza incidence in various regions of China (9), few have explored the spatiotemporal patterns in plateau areas like Qinghai, where altitude and geographic features may influence the dynamics of influenza spread. Therefore, this study employed influenza surveillance data from Qinghai Province to analyze the county - level spatial heterogeneity of influenza incidence, explore the spatiotemporal distribution and evolution trends in the plateau region, and identify high - incidence areas. The aim is to offer theoretical and practical support for influenza surveillance, early warning, risk assessment, and precise control in this region.

## Methods

### Data and sources

The influenza occurrence data of 45 counties in Qinghai spanning from 2009 to 2023 were collected through the China Infectious Disease Surveillance System, guaranteeing the credibility of the data. The map data were sourced from the Geological Survey Institute of Qinghai Province. The map employed in this research features a scale of 1:100,000,0, presenting a detailed display of the region’s administrative divisions.

### Spatial rank correlation

Utilized ArcGIS software to create thematic maps of influenza incidence in Qinghai Province. Moreover, calculated the global Moran’s I to describe the spatial characteristics of influenza incidence in Qinghai Province. Equations 1, 2 are shown as follows:


(1) $$s^{2}=\frac{\Sigma_{i=1}^{n}\left(x_{i}-\bar{x}\right)}{n}$$

(2) $$I=\frac{\sum_{i=1}^{n}\sum_{j=1}^{n}w_{ij}\left(x_{i}-\bar{x}\right)\left(x_{j}-\bar{x}\right)\;}{s^{2}\Sigma_{i=1}^{\pi }\Sigma_{j=1}^{n}w_{ij}}$$

- n: Represents the number of counties;
- x: Represents the influenza incidence rates of different counties;
-
$\bar{x}$ : Represents the mean incidence rate;
- w: Represents spatial weight;
-
$w_{ij}$ :Represents an element in the spatial weight matrix, indicating the spatial relationship weight between locations i and j.

Moran’ s I falls within the range of −1 to 1. When the significance level is set at *α* = 0.05, values close to 1 signify positive spatial autocorrelation, those near −1 imply negative spatial autocorrelation, and zero represents randomness. We employed Local Moran’s I to examine spatial autocorrelation patterns at the spatial unit level and carried out LISA to pinpoint clustered areas. Four clustering patterns were detected: HH (high-high), HL (high-low), LL (low-low), and LH (low-high). The Getis - Ord G statistic was used as a measure to identify influenza incidence hotspots and cold spots by using the Equation 3:


(3) $$G_{i}=\overset{n}{\underset{j=1}{\sum }}w_{ij}x_{j}∕\overset{n}{\underset{j=1}{\sum }}xj$$

In Equation 3, the meanings of all variables are the same as those in Equation 1. Under the test level of *α* = 0.05, a positive Gi value indicates an area with a high incidence of influenza, while a negative Gi value indicates an area with a low incidence of influenza.

### Gravity center shift model and standard deviation ellipse

The gravity - center shift model is commonly used to analyze the gravity - center position of a certain attribute in geospatial data and its changing trends over time. In this study, the influenza incidence gravity - center model was adopted to calculate the coordinates of the influenza incidence gravity - center in different periods. By calculating the gravity - center coordinates in different periods, we can observe their moving directions and distances, thus visually presenting the overall distribution center of the research object in space, as well as its moving directions and distances. The Equation 4 for calculating the gravity center is as follows: Here, $x_{i}$ and $y_{i}$ represent the coordinates of the i -th county, while $w_{i}$ represents the weight of the i -th county. In this study, the weight is defined as the influenza incidence rate.


(4) $$\bar{X}=\frac{\Sigma w_{i}x_{i}}{\Sigma w_{i}}\bar{Y}=\frac{\Sigma w_{i}y_{i}}{\Sigma w_{i}}$$

Some simple spatial analysis methods, such as density analysis, can only reflect the degree of data concentration and cannot embody characteristics like direction. The standard deviational ellipse method has unique advantages in terms of simplicity, intuitiveness, and the ability to comprehensively describe spatial distribution characteristics. The standard deviational ellipse method calculates the standard deviation of data points relative to the mean value and constructs an elliptical shape, which can intuitively present the spatial distribution pattern of the data. In this way, it can display the degree of data concentration, distribution direction, and degree of dispersion in space. In this study, the size of the ellipse mirrors the degree of concentration of influenza incidence reports in Qinghai on a spatial scale. The length of the major axis shows the direction in which the data are distributed, whereas the length of the minor axis represents the extent of data distribution. The larger the difference between the lengths of the major and minor axes (that is, the greater the eccentricity), the more distinct the directionality of the data. On the contrary, if the lengths of the major and minor axes are more similar, it implies that the directionality is less distinct.

### Statistical analysis

GraphPad Prism 10 software was utilized for trend analysis and the creation of line graphs. Meanwhile, ArcGIS 10.8 software served for map visualization and spatial analysis purposes. The spatial exploratory analysis involved several aspects, including global spatial autocorrelation analysis, local spatial autocorrelation analysis, and hotspot analysis. As for spatial descriptive analysis, it was conducted by applying the methods of gravity center analysis and standard deviation ellipse analysis.

## Results

### Incidence characteristics

**Figure 1:**
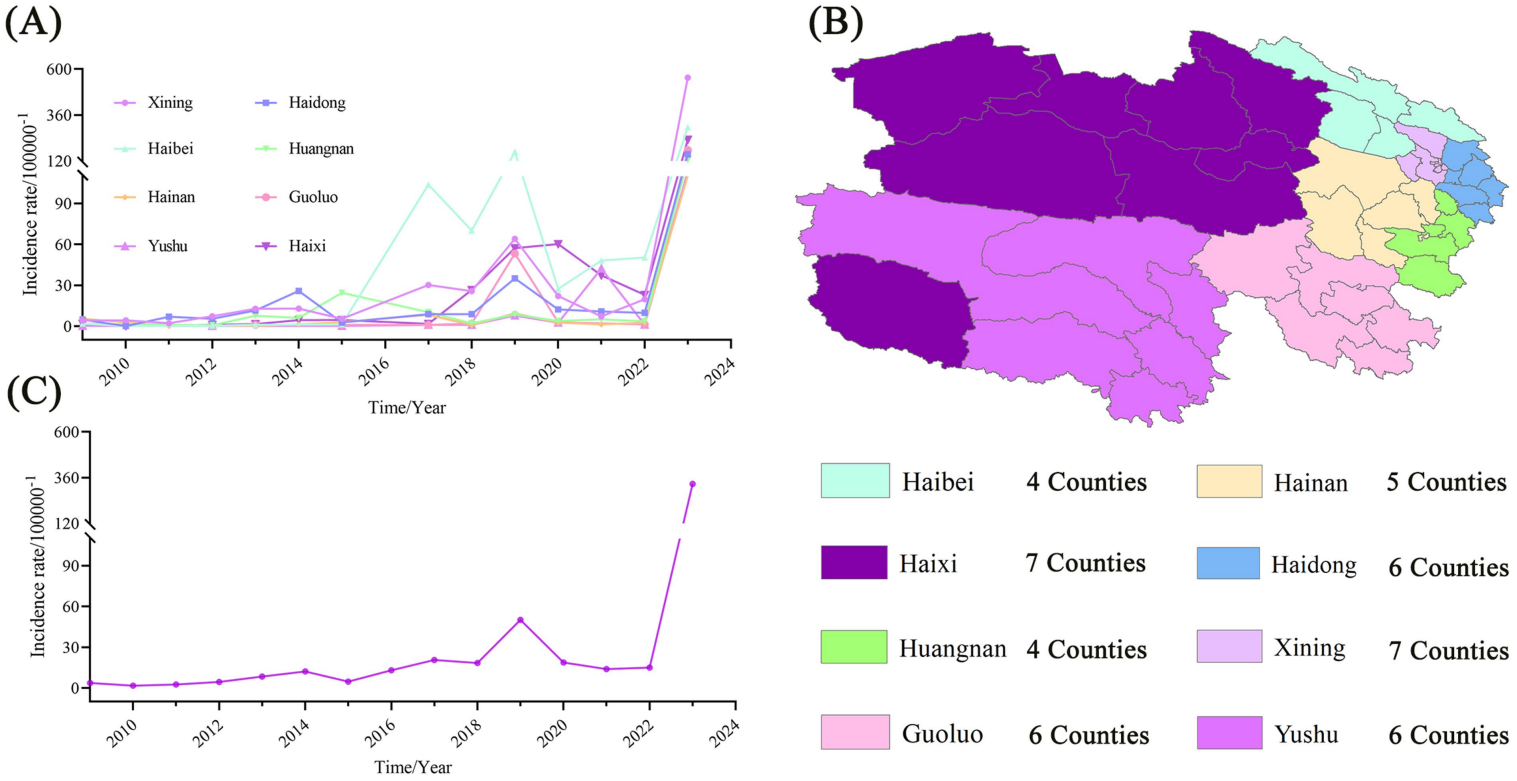
Trend chart of influenza incidence in different regions of Qinghai Province. **(A)** Trend of influenza incidence from 2009 to 2023 in 8 cities of Qinghai Province. **(B)** Regional division of cities in Qinghai. **(C)** Average incidence of influenza in Qinghai Province from 2009 to 2023.

From 2009 to 2023, a total of 30,639 influenza cases were reported in 45 counties of Qinghai Province. During these 15 years, the annual incidence rate fluctuated significantly. It dropped to as low as 1.74 per 100,000 in 2010, rose to 50.16 per 100,000 in 2019, and skyrocketed to 327.19 per 100,000 in 2023. The incidence trends in each prefecture - level city and the average incidence trend are shown in Figure 1.

**Figure 2:**
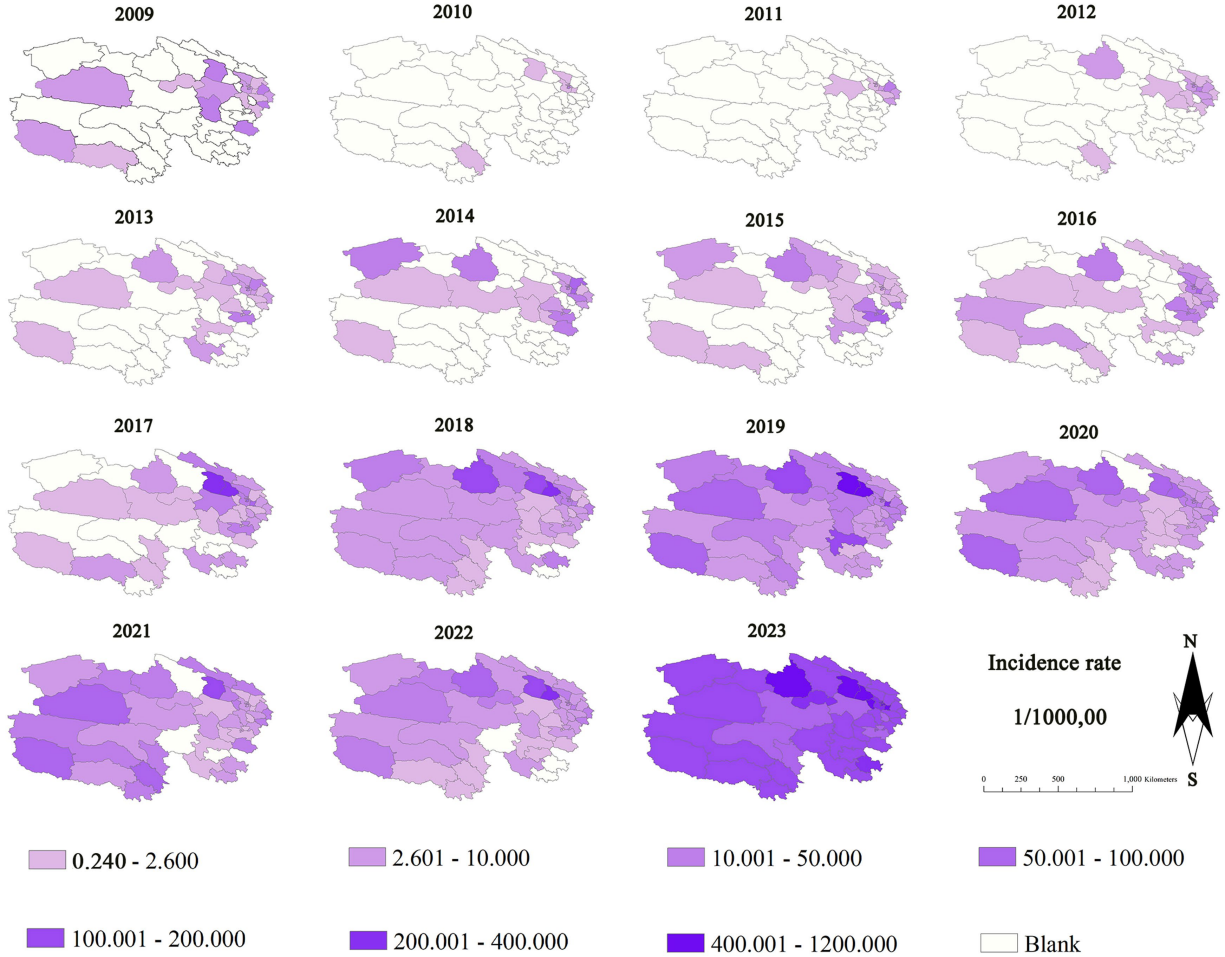
Thematic map of influenza incidence from 2009 to 2023 in each county of Qinghai Province.

From 2009 to 2017, the incidence rates in various counties of Qinghai were generally lower than 10 per 100,000, especially in the western region, where the changes were minimal. From 2018 to 2021, the incidence rates in the eastern counties steadily increased to over 50 per 100,000 and then declined. In 2023, the incidence rate of the whole province rose significantly again, and the eastern region was greatly affected. The incidence rates in the five counties of Xining (Chengdong, Chengzhong, Chengxi, Chengbei, Huangyuan), the three counties of Haidong (such as Ping’an), and Delingha City exceeded 400 per 100,000. The detailed results are shown in Figure 2.

### Spatial correlation analysis

The global spatial autocorrelation analysis of influenza incidence rates in 45 counties of Qinghai shows that the incidence rates from 2009 to 2011 and 2015 exhibited negative spatial autocorrelation, but only 2015 was statistically significant, and that the incidence rates from 2012 to 2014 and from 2016 to 2023 exhibited positive spatial autocorrelation, but the rates from 2012 to 2014, 2016 and 2021 were not statistically significant. The strongest clustering occurred in 2023, followed by 2018. Detailed results are presented in Table 1.

**Table 1:** Global spatial autocorrelation of influenza incidence from 2009 to 2023 in Qinghai (1/100,000).

Year	Rate	Moran’s I	*Z*	*P*
2009	3.807	−0.185	−0.639	0.263
2010	1.741	−0.078	0.277	0.423
2011	2.630	−0.061	0.329	0.363
2012	4.471	0.086	0.779	0.186
2013	8.497	0.279	1.394	0.098
2014	12.236	0.166	1.116	0.135
2015	4.731	−0.215	−2.190	0.010
2016	13.035	0.057	0.537	0.251
2017	20.675	0.444	3.969	0.014
2018	18.433	0.321	3.299	0.017
2019	50.165	0.309	3.370	0.012
2020	18.853	0.195	1.790	0.050
2021	13.994	0.177	1.695	0.063
2022	15.084	0.329	4.110	0.008
2023	327.195	0.447	3.818	0.002

**Figure 3:**
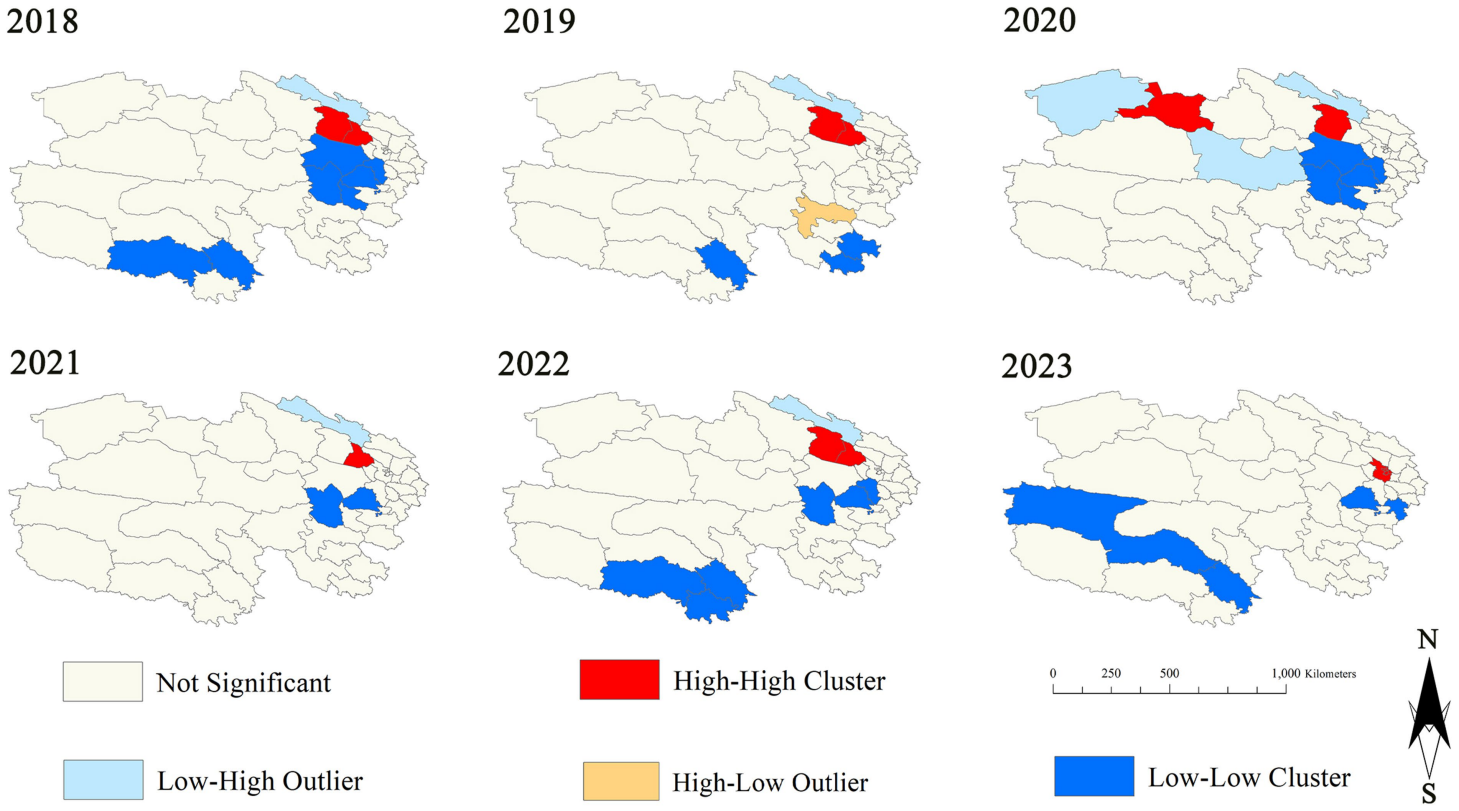
The LISA cluster map of influenza incidence in Qinghai region from 2018 to 2023.

The local spatial autocorrelation analysis was conducted for influenza incidence rates from 2018 to 2023, which exhibited positive spatial autocorrelation. Significant clustering patterns were identified. In eastern and northern Qinghai, the predominant patterns were high-high clustering and low-high clustering, while in western and southern Qinghai, low-low clustering predominated. The number of high-high clustering patterns observed each year increased from 2 to 5, indicating a strengthening of local spatial relationships and an increased influence of influenza incidence rates among counties. See Figure 3 for details.

### Hotspot analysis

**Figure 4:**
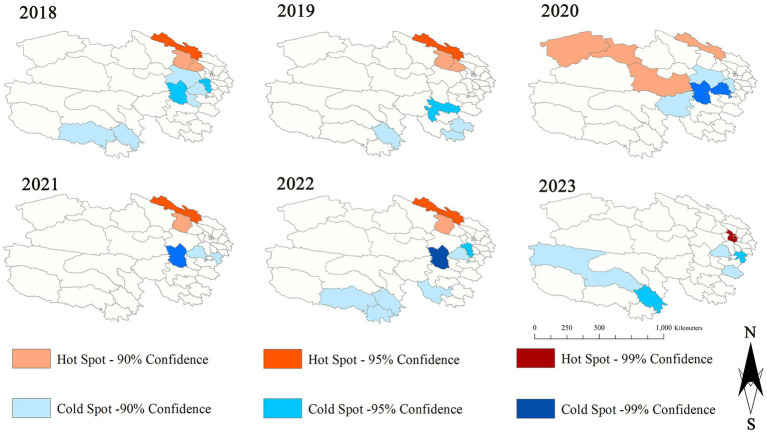
Hot sports of influenza incidence from 2018 to 2023 in Qinghai.

The results of the hotspot analysis of influenza incidence in 2018–2023 showed that hotspot areas were identified in 3, 3,4,2, 2 and 5 counties for each respective year. These hotspots were mainly concentrated in eastern Qinghai, particularly in two regions: Xining and Haibei. Additionally, the fluctuations in the number of hotspot counties are not significant, and all are dominated by eastern Qinghai. See Figure 4 for detailed information.

## Standard deviation ellipse analysis and gravity center migration

The standard deviation ellipses were centered around the influenza incidence gravity center for each respective year. In 2021, the standard deviation ellipse covered the largest area, encompassing almost all of eastern Qinghai and parts of western Qinghai, indicating widespread transmission. From 2018 to 2021, the standard deviation ellipse gradually elongated amid fluctuations, reflecting a gradual expansion of the influenza transmission range. From 2021 to 2022, the standard deviation ellipse shrank significantly, indicating a more localized spread. In 2023, the standard deviation ellipse increased again, reflecting another expansion of influenza transmission. From 2018 to 2023, influenza was mainly concentrated in the eastern part of Qinghai, indicating a clear clustering effect of influenza incidence in this region.

**Figure 5:**
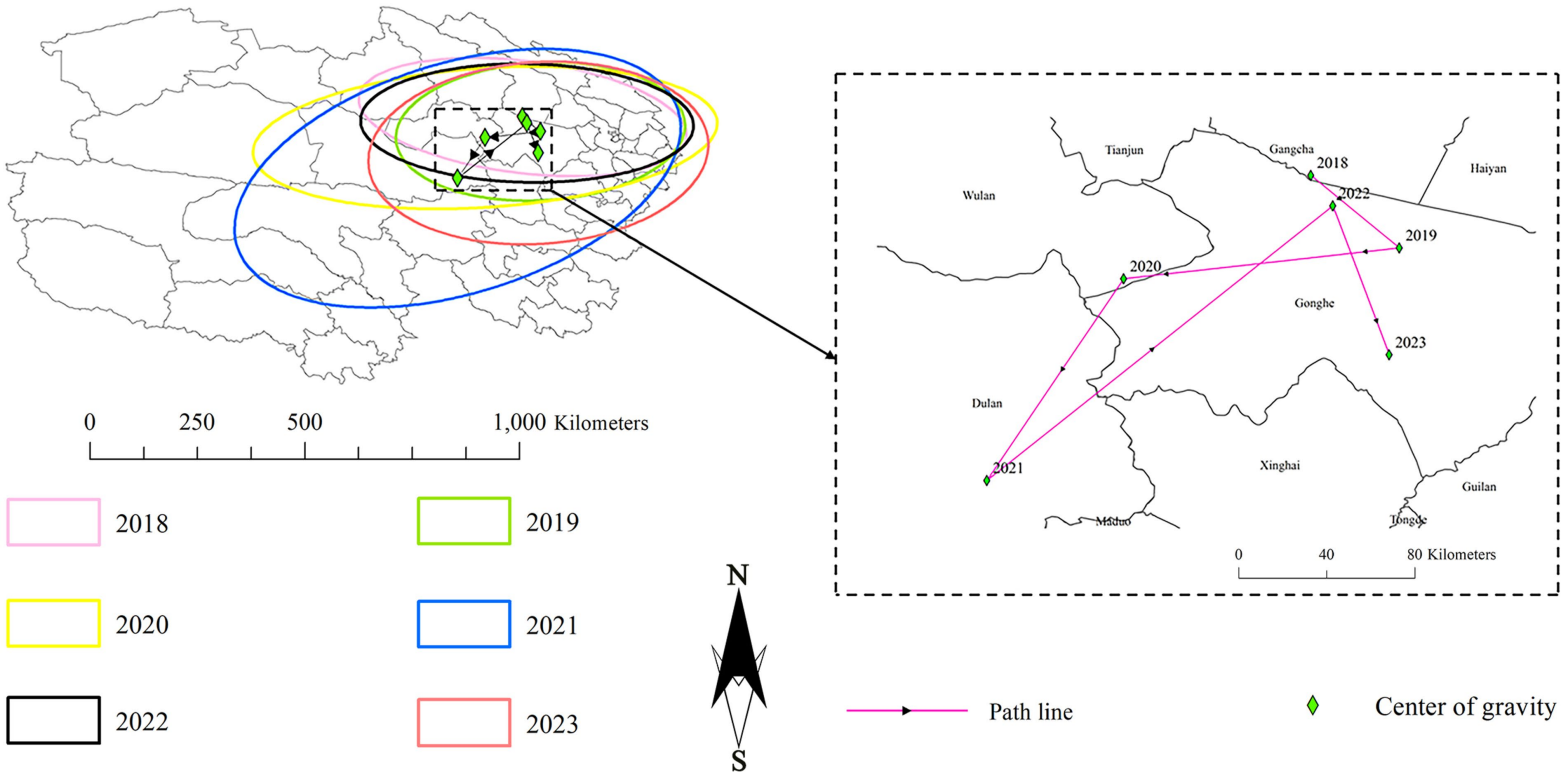
Gravity center of influenza incidence from 2018 to 2023 in Qinghai.

The trajectory of Qinghai’s influenza incidence gravity center from 2018–2023 reflects the regional influenza spread. In 2018, it was in Gangcha County, northern - eastern Qinghai. In 2019, it shifted 49.2 km east to Gonghe County, showing an eastward spread. In 2020, it moved 102.9 km southwest to Wulan County, expanding westward. In 2021, it went 101.5 km south to Dulan County, with southern incidence rising. In 2022, it moved 174.7 km back to Gonghe County, indicating epidemic fluctuations. In 2023, it stayed in Gonghe but shifted 69.8 km southeast, with high - incidence in eastern Qinghai moving south. Overall, from 2018–2023, the gravity center remained in eastern Qinghai, moving 86.5 km southeast from Gangcha to Gonghe, and shifted around Xining and its surroundings annually. See Figure 5 for details.

## Discussion

Spatiotemporal analysis techniques offer substantial benefits in uncovering the development trajectories of influenza epidemics and precisely forecasting associated risks (10). Through the examination of influenza’s dynamic shifts across both time and space, high - risk zones and potential trends can be accurately pinpointed (11). Conducting in - depth research at the county level is crucial for enhancing the accuracy and efficiency of influenza prevention and control measures. This study delves into the spatiotemporal evolution of influenza incidence at the county level in Qinghai, thereby emphasizing the vital role and practical value of spatiotemporal analysis in epidemic prevention and control efforts of plateau region.

It was found by us that during the period from 2009 to 2023, the incidence rate of influenza in Qinghai witnessed significant fluctuations and showed an overall upward trend. During the period from 2009 to 2015, the incidence rate stayed at a low level, which was below 15 per 100,000. The relatively low incidence of influenza during this period is likely related to the fact that the reporting of notifiable infectious diseases was in its initial stage in the early period. In the early years in Qinghai, primary - level medical units suffered from insufficient testing equipment and a shortage of professional staff. This led to the omission of influenza cases in some areas during the detection and reporting process. As a result, the incidence data obtained from some areas in the initial stage of the research may have underestimated the actual scale of influenza incidence. From 2016 to 2019, the incidence rate of influenza showed a gradually increasing trend. Especially in 2019, it reached the peak (50.17 per 100,000). Compared with the previous six years, the increase in the incidence rate during this period was 369.77%. From 2020 to 2022, the influenza incidence rate dropped significantly, ranging from 15.08 to 18.85 per 100,000. The possible reason is that this period coincided with the outbreak of COVID - 19 (12), and the population prevention and control measures were relatively strict (13), thus leading to a decrease in the incidence rate of influenza.

However, in 2023, the influenza incidence rate rebounded sharply to 327.19 per 100,000. This represented a 552.24% increase compared to 2019 and a 2069.12% increase compared to 2022. This further demonstrates that the prevention and control measures related to the COVID - 19 pandemic are conducive to suppressing the incidence rate of influenza. We also found that from 2009 to 2023, the average incidence rates in Haibei Prefecture (with an average altitude of 3,655 meters) and Hainan Prefecture (with an average altitude of 3,531 meters) were 59.27 and 10.94% respectively, ranking highest and lowest in terms of the flu incidence rate in Qinghai Province. The incidence of influenza in Qinghai Province generally shows a pattern of lower incidence in the west and higher in the east, which is exactly opposite to the province’s terrain of higher altitude in the west and lower in the east. Overall, the higher the altitude, the lower the incidence.

The significant differences in influenza incidence between western and eastern Qinghai may be attributed to various social factors, including population density, medical resources, altitude, transportation accessibility (14), and the perfection of the influenza reporting system. Some studies indicate that in areas with higher population density and better transportation networks, the influenza transmission rate tends to be higher (14, 15). Some studies also suggest that lower temperatures can facilitate the spread of influenza viruses by enhancing their stability and transmission (16, 17). Therefore, in subsequent research, social factors affecting the transmission of influenza and the nature of the influenza virus itself can be considered for inclusion.

We further conducted a global spatial autocorrelation analysis. The results showed that the influenza incidence rate in Qinghai exhibited positive spatial correlation in 2015, 2017–2019 and from 2022 to 2023. Distinct clustering patterns in different regions of Qinghai were observed. High - high clusters (HH) are mainly distributed in Gangcha, Xining, Dachaidan and other places. High - low clusters (HL) are distributed in Gande and other places. Low - low clusters (LL) are distributed in Yushu, Hainan and other places. The HH in these regions indicates that counties with a high incidence of influenza are surrounded by other such counties, suggesting strong local transmission (18). The LH means that counties with a low incidence of influenza are encircled by those with a high incidence, implying potential risk areas. We also found that the altitude of the areas where HH and LH are distributed is concentrated between 3,000 and 3,500 (meters). Low - low clusters and high - low clusters are mainly distributed at an altitude between 3,500 and 4,500 meters, indicating that the disease incidence risk in these areas is relatively low. Overall, there is a phenomenon that the lower the altitude, the higher the risk of influenza incidence.

We further verified the authenticity of this result through the hot-spot map. The findings revealed that from 2018 to 2023, all the hot - spot counties in Qinghai Province were mainly concentrated in areas at an altitude of 3,000–3,500 meters, such as Qilian, Mangya, Xining, Gangcha, and Haiyan. This is basically consistent with the results of cluster analysis.

Through standard deviational ellipse analysis and centroid migration, it was found that from 2018 to 2023, the centroid of influenza incidence remained in the eastern region of Qinghai Province, mainly shifting among the four counties of Gangcha, Wulan, Dulan, and Gonghe. The incidence rate in the eastern part of Qinghai Province increased relatively rapidly, causing the overall centroid to move from the southeast to the northwest. The standard deviational ellipse was in a southwest - northeast pattern, and its area fluctuated over time, reflecting the degree of concentration of influenza incidence. These results further indicate a significant difference in incidence between the eastern and western parts of Qinghai. Overall, the incidence in the eastern part of Qinghai Province is significantly higher than that in the western part, making it a hot - spot and major area for the migration of the disease center. The spread of influenza shows a continuous expanding trend in the northern and eastern parts of Qinghai Province. From 2018 to 2023, the center of influenza incidence has gradually shifted toward Xining, Haidong, and their surrounding areas. Especially in 2023, the influenza epidemic in Xining was more concentrated. For the eastern region of Qinghai Province, which serves as a hot - spot and severely - affected area for influenza incidence, continuous efforts should be made to strengthen the reserve of medical resources. For instance, increasing the number of influenza vaccine vaccination sites and expanding the stockpile of antiviral drugs. In the western region, due to the relatively low incidence rate and weak medical resources, it can draw on the prevention and control experience of the eastern region. It is necessary to make advance plans for material reserves, and enhance medical cooperation and exchanges with the eastern region, so as to improve the ability to respond to sudden influenza outbreaks. In the northern region, as the epidemic shows an expanding trend, it is essential to make early - stage arrangements. This includes strengthening the construction of public health infrastructure, enhancing the prevention and control management of key places such as schools and nursing homes. Meanwhile, extensive health publicity and education should be carried out to raise the public’s awareness of prevention and control.

In light of the altitude differences, efforts should be made to strengthen influenza prevention and control work in Qinghai Province, to improve the influenza monitoring capabilities, to properly address the issue of medical resource allocation between the two regions, and to better respond to the public health challenges posed by influenza epidemics.

Based on the above results, for influenza prevention and control in Qinghai Province, it is necessary to formulate differentiated and precise prevention and control strategies according to the aggregation patterns and spatiotemporal characteristics of different regions (19). The eastern part of Qinghai Province, with a relatively low altitude, is a high - incidence area for influenza and requires more prevention and control resources to address this challenge. Measures such as increasing vaccine supply, strengthening the construction of medical facilities, and improving the professional capabilities of medical staff are necessary (20). In addition, targeted health education activities should be carried out to enhance the public’s awareness and prevention of influenza (21). Although the influenza incidence in the western part of Qinghai Province is relatively low, there are still certain risks. Moreover, due to the high altitude in this region, a more comprehensive high - altitude influenza monitoring system needs to be established to detect and respond to potential influenza epidemics in a timely manner. In light of the altitude differences, efforts should be made to strengthen influenza prevention and control work in Qinghai Province, to improve the influenza monitoring capabilities, to properly address the issue of medical resource allocation between the two regions, and to better respond to the public health challenges posed by influenza epidemics.

## Conclusion

The incidence of influenza in Qinghai Province demonstrates remarkable spatiotemporal heterogeneity. Temporally, the incidence rate experiences significant fluctuations, with an overall upward trajectory. Spatially, the influenza incidence is higher in the eastern region and lower in the western region. The spread of influenza across the province emanates from the eastern region as the core, and the epicenter of the epidemic has been gradually shifting toward areas such as Xining and Haidong and their peripheries. Consequently, it is imperative for Qinghai Province to increase the investment in prevention and control resources in the eastern region, improve the high - altitude monitoring system in the western region, optimize the allocation of medical resources, and enhance the overall influenza prevention and control capabilities of the entire province.

## Limitations

### Limitations of the influenza reporting system

The influenza reporting system in Qinghai mainly relies on the reporting from medical institutions. However, in the early years, due to insufficient testing equipment and a shortage of professional staff in primary - level medical units, influenza cases in some areas might have been missed during detection and reporting. These limitations led to the fact that the incidence data obtained from some areas in the early stage of the research might have underestimated the actual scale of influenza incidence. As a result, it was impossible to accurately present the real - time spread of influenza across the province in the early years.

### Lack of risk factor analysis

This study did not fully analyze the impacts of risk factors such as population density, climatic conditions, and residents’ hygiene habits on influenza incidence. The lack of risk factor analysis makes it difficult to comprehensively understand the pathogenesis and transmission patterns of influenza in Qinghai. Consequently, the research results have deficiencies in explaining the differences in incidence among different regions and predicting the development of the epidemic.

## Data Availability

The datasets presented in this study can be found in online repositories. The names of the repository/repositories and accession number(s) can be found in the article/Supplementary material.
